# A Transcriptome Study of Progeroid Neurocutaneous Syndrome Reveals POSTN As a New Element in Proline Metabolic Disorder

**DOI:** 10.14336/AD.2018.0222

**Published:** 2018-12-04

**Authors:** Yu-Wen Huang, Ming-Fu Chiang, Che-Sheng Ho, Pi-Lien Hung, Mei-Hsin Hsu, Tsung-Han Lee, Lichieh Julie Chu, Hsuan Liu, Petrus Tang, Wailap Victor Ng, Dar-Shong Lin

**Affiliations:** ^1^Institute of Biotechnology in Medicine and Department of Biotechnology and Laboratory Science in Medicine, National Yang Ming University, Taipei, Taiwan.; ^2^Department of Medical Research, Mackay Memorial Hospital, Taipei, Taiwan.; ^3^Department of Neurosurgery, Mackay Memorial Hospital, Taipei, Taiwan.; ^4^Mackay Junior College of Medicine, Nursing and Management, Taipei, Taiwan.; ^5^Graduate Institute of Injury Prevention and Control, Taipei Medical University, Taipei, Taiwan.; ^6^Department of Pediatrics, Mackay Memorial Hospital, Taipei, Taiwan.; ^7^Department of Pediatric Neurology, Kaohsiung Chang Gung Memorial Hospital, and Chang Gung University College of Medicine, Kaohsiung, Taiwan.; ^8^Molecular Medicine Research Center, Chang Gung University, Taoyuan, Taiwan.; ^9^Department of Cell and Molecular Biology, College of Medicine, Chang Gung University, Taoyuan, Taiwan.; ^10^Molecular Regulation and Bioinformatics Laboratory and Department of Parasitology, Chang Gung University, Taoyuan, Taiwan.; ^11^Institute of Biomedical Informatics and Center for Systems and Synthetic Biology, National Yang Ming University, Taipei, Taiwan.; ^12^Department of Biochemistry, Kaohsiung Medical University, Kaohsiung, Taiwan.; ^13^Department of Medicine, Mackay Medical College, New Taipei, Taiwan

**Keywords:** Aging, cutis laxa, progeroid, PYCR1, periostin, ARCL2B

## Abstract

Aging is a complex biological process. A study of pyrroline-5-carboxylate reductase 1 (PYCR1) deficiency, which causes a progeroid syndrome, may not only shed light on its genetic contribution to autosomal recessive cutis laxa (ARCL) but also help elucidate the functional mechanisms associated with aging. In this study, we used RNA-Seq technology to examine gene expression changes in primary skin fibroblasts from healthy controls and patients with *PYCR1* mutations. Approximately 22 and 32 candidate genes were found to be up- and downregulated, respectively, in fibroblasts from patients. Among the downregulated candidates in fibroblasts with *PYCR1* mutations, a strong reduction in the expression of 17 genes (53.1%) which protein products are localized in the extracellular space was detected. These proteins included several important ECM components, periostin (POSTN), elastin (ELN), and decorin (DCN); genetic mutations in these proteins are associated with different phenotypes of aging, such as cutis laxa and joint and dermal manifestations. The differential expression of ten selected extracellular space genes was further validated using quantitative RT-PCR. Ingenuity Pathway Analysis revealed that some of the affected genes may be associated with cardiovascular system development and function, dermatological diseases and conditions, and cardiovascular disease. *POSTN*, one of the most downregulated gene candidates in affected individuals, is a matricellular protein with pivotal functions in heart valvulogenesis, skin wound healing, and brain development. Perturbation of PYCR1 expression revealed that it is positively correlated with the POSTN levels. Taken together, POSTN might be one of the key molecules that deserves further investigation for its role in this progeroid neurocutaneous syndrome.

Aging is a complex biological process that results in systemic physiological changes, particularly in the skin and the cardiovascular, skeletal, and neuronal systems. Autosomal recessive cutis laxa (ARCL) includes several connective tissue disorder syndromes characterized by abnormal elastic fibers, resulting in sagging, inelastic, and wrinkled skin. It involves multiple organ systems, leading to multisystem disorder characterized by premature aging. It may be used as a human disease model that mimics physiological aging. ARCL type 1 (ARCL1; MIM 219100) is caused by defects in ECM components, and mutations have been identified in the following genes: EGF-containing fibulin-like ECM protein (*EFEMP2 *or *FBLN4*), fibulin 5 (*FBLN5*), and latent transforming growth factor-beta-binding protein-4 (*LTBP4*). Unlike ARCL1, ARCL2 (ARCL2A and ARCL2B; MIM 219200 and MIM 612940) is caused by mutations in genes involved in different metabolic pathways, including glycosylation (ATPase H^+^ transporting V0 subunit A2 (*ATP6V0A2*); ARCL2A), proline biosynthesis (pyrroline-5-carboxylate reductase 1 (*PYCR1*); ARCL2B), and Golgi apparatus function (RAB6-interacting golgin (*GORAB*)). In addition to connective tissue abnormalities, a spectrum of syndromes with growth and developmental delay, joint laxity, and mental retardation are also found in most ARCL2B patients [1-4]. Previous study showed *PYCR1 *mutations leads to irregular, fragmented, and reduced elastic fibers in the reticular layer of patients skin [5, 6]. However, the mechanism through which metabolic enzyme deficiency causes connective tissue abnormality is largely unknown.

Proline and its derivative, hydroxyproline, play an essential role in the formation of collagen and elastin. PYCR1 or Δ^1^-pyrroline-5-carboxylate synthase (P5CS) defect leads to decreased in mitochondrial proline synthesis [2, 7, 8]. PYCR1 is a member of the PCYR family, which includes PYCR1, PYCR2, and PYCRL. PYCRL is a cytoplasmic enzyme, and PYCR1 and PYCR2 are mitochondrial proteins that catalyze the reduction of Δ^1^-pyrroline-5-carboxylate to proline, which is the final step in the synthesis of proline from glutamate (Fig. 1A). Moreover, PYCR1 is the most highly expressed in the bone and skin. *PYCR1 *gene mutations may result in ARCL2B. Approximately 60 individuals in more than 30 families have been reported to have PYCR1-related ARCL with progeroid features [5, 6, 9-14]. PYCR1 deficiency leads to a premature aging phenotype, intrauterine growth retardation, a characteristic triangular facial gestalt, psychomotor retardation, hypotonia, and ophthalmologic abnormalities; and progeroid cutaneous manifestations are the most relevant distinctive hallmarks [9]. Although studies have focused on the genotype-phenotype correlation, the link between PYCR1 deficiency and connective tissue and ophthalmologic changes remain unexplored.

Studies investigating the clinical applications of next-generation sequencing technology have started a new era of molecular genetic diagnosis. The global gene expression profiles of *PYCR1* in primary skin fibroblasts from healthy controls and patients with *PYCR1* mutations were compared using RNA-Seq analysis. Our results provided a comprehensive understanding of how *PYCR1* mutations may cause connective tissue abnormalities, preglaucoma, and heart valve disease. This work not only helps elucidate the functional mechanisms associated with aging but also provides the first characterization of the transcriptomic basis for *PYCR1* mutations causing autosomal recessive neurocutaneous syndrome

## MATERIALS AND METHODS

### Patients

Four patients (P1, P2, P3, and P4), aged 4 to 9 years old, from three families with features of ARCL2B were found to have, *PYCR1* mutations by DNA sequencing using the BigDye terminator chemistry in this (P4) and our previous (P1, P2, and P3) studies [11, 12]. These patients presented with generalized skin wrinkling, intrauterine growth retardation, a typical facial gestalt, and variable CNS involvement. Consent for molecular studies was given by all individuals involved in this study or their legal representatives. This study was approved by the Ethics Committee of Mackay Memorial Hospital (14MMHIS046).

### Cell cultures

Using standard techniques, skin fibroblasts were isolated and cultured from skin biopsies of the patients and age-matched healthy controls (3 to 8 years old). Skin fibroblasts were cultivated in DMEM (Gibco, Bethesda, MD, USA) supplemented with 10% fetal bovine serum (FBS; Biological Industries, Kibbutz Beit-Haemek, Israel), 1% glutamine (Gibco), and 1% penicillin/streptomycin (Gibco) and harvested when the cells reached approximately 100% confluency (Passage: 6 (RNA-Seq); 6 to 9 (other experiments)).

### Transcriptome sequencing

RNA-Seq analysis were conducted using total RNA samples from two controls and two patients (P1 and P3) isolated with Trizol (Gibco). An aliquot of 2.5 μg of total RNA with rRNA removed using Ribo-Zero™ Gold Kit (Epicentre, Madison, WI, USA) was fragmented with RNase III (Thermo Fisher Scientific, Waltham, MA, USA). The yield of fragmented RNA was quantitated using the Quant-iT RNA assay kit (Invitrogen, Carlsbad, CA, USA). After following the protocol for the RiboMinus™ kit (Thermo Fisher Scientific), 50-ng ribominus RNA fragments were used for transcriptome libraries preparation as per the manufacturer’s protocol and were amplified using the 5500 W Barcode Conversion Primer Kit for 5500 SOLiD System (Life Technologies, Gaithersburg, MD, USA). The bar-coded cDNA libraries were pooled together in equal concentrations and were sequenced in the same quadrant of a slide on a SOLiD sequencer (Applied Biosystems, Foster City, CA, USA) for whole-transcriptome sequencing.

### RNA-Seq data analysis

The high-throughput sequencing of four samples (two healthy controls and two patients, P1 and P3) was performed using single-end, 150 nucleotide reads from the 5500 SOLiD System (Life Technologies). All quality color-space reads were cut to 50 nucleotides and mapped against the NCBI Build 37/hg19 genome using LifeScope Genome Analysis Software v2.5.1 with default RNA-Seq parameters (Life Technologies). Expression values of each gene in TPM (transcripts per million) were determined and statistical analysis performed in the RNA-Seq workflow of Partek Genomics Suite (v6.6, St. Louis, MO, USA). Gene Ontology and Function analysis were performed using the PANTHER Classification System (www.pantherdb.org/) [15, 16] and Ingenuity® Pathway Analysis (IPA) software (www.ingenuity.com), respectively.

### Quantitative RT-PCR (qRT-PCR)

Fibroblast total RNAs were isolated using an RNeasy Mini Kit (QIAGEN, Hilden, Germany) according to the manufacturer’s procedure. Complementary DNAs were synthesized by the reverse transcription of 1 µg of total RNAs by using a RevertAid H Minus First Strand cDNA Synthesis Kit (Thermo Fisher Scientific). qPCR was performed using a Fast SYBR® Green Master Mix (Applied Biosystems) on an ABI Prism 7500 (Applied Biosystems) with gene-specific primers (Supplementary Table 2). For data analysis, raw threshold cycle (CT) values were normalized to *GAPDH* to obtain ΔCT. Normalized ΔCT was calibrated to the control cell samples for ΔΔCT.

### Proline assay

Fibroblasts cultured in DMEM containing 5% FBS for 3 days were harvested and lyzed through sonication after resuspension in 1X PBS. The cell lysates were centrifuged at 15,000 *g* at 4°C for 10 min, and the proteins in the supernatant were depleted by incubation with a final concentration of 10% trichloroacetic acid on ice for 5 min and were centrifuged as described earlier. The proline concentration in the protein-depleted supernatant was determined according to the method of Wang et al. [17]. Briefly, 0.5 mL each of glacial acetic acid and Chinard’s reagent (2.5 g of ninhydrin dissolved in 60 mL of glacial acetic and 40 mL of 6 M orthophosphoric acid at 70°C) were added to 250 μL of the supernatant and incubated for 10 min at 90°C. The proline concentration was determined colorimetrically at 515 nm and was calculated using the proline calibration curve.

### Western blot analysis

An aliquot of 20 μg of proteins was separated on 10% or 12% SDS-PAGE and transferred to PVDF membranes (Amersham-Pharmacia Biotech Inc., Buckinghamshire, UK). After the membranes were blocked with 5% skim milk in TBST buffer (25 mM Tris-HCl, 137 mM NaCl, and 0.1% Tween 20, pH 7.4) for 1 h, the membranes were incubated with rabbit anti-human PYCR1(Cat. no. 13108-1-AP), PYCR2 (Cat. no. 17146-1-AP) (Proteintech), and POSTN (Cat. no. ab152099, Abcam) polyclonal antibodies, and mouse anti β-actin (Cat no. A2228) and α-tubulin (Cat. no. T6199) (Sigma) monoclonal antibodies at 4°C overnight. After the membranes were washed three times with TBST, the membranes were incubated with horseradish peroxidase (HRP)-conjugated [goat anti- rabbit or -mouse] IgG secondary antibody (1: 5,000-1: 10,000) at room temperature for 1 h, and the immune reactions were detected using an ECL detection system (Immobilon Western Chemiluminescent HRP Substrate, Millipore, MA, USA).

### Knockdown of PYCR1 in fibroblasts

Five recombinant shRNAs for the *PYCR1* gene (shPYCR1 clone IDs: TRCN0000038979, -80, -81, -82, and -83) and TRC1 obtained from the National RNAi Core Facility at Academia Sinica, Taipei, Taiwan were subcloned into the pLKO plasmid to obtain shPYCR1 #1 to #5 and the scramble control. For silencing PYCR1 in primary skin fibroblasts, shPYCR1-#1-#5 lentiviruses were generated by transfecting 293T cells with the recombinant DNAs and the lentiviral helper plasmids by using the TransIT-LT1 in vitro transfection reagent (Mirus, Madison, WI, USA). After removing the cell debris in the supernatant by centrifugation according to the recommended procedure, lentiviruses were harvested 48 h post-transfection by filtration through 0.22-µm cellulose acetate filters. Subsequently, skin fibroblasts (10^5 ^cells/well in a 6-well plate) were cultured in a medium containing 8 µg/mL polybrene, infected with the lentiviruses (MOI = 2 or 5), and incubated at 37°C with 5% CO_2_ according to the recommended procedure. Selection of the infected cells began on day 3 with medium containing 1 µg/mL puromycin, which was replaced every 3 days.

### Overexpression of PYCR1 in fibroblasts

The PYCR1 overexpression plasmid was constructed by cloning a 1001-bp cDNA fragment, which consisted of the 960-bp CDS of *PYCR1* (Acc. no. NM_001282280), and *Bam*HI and *Eco*RI adaptors, into a lentiviral vector pCDH-CMV-MCS-EF1-Puro (System Biosciences, Mountain View, CA). The cDNA fragment was prepared by PCR amplification of a cDNA library originating from healthy human skin fibroblasts with forward and reverse primers (Supplementary Table 1) by using the high-fidelity Phusion enzyme (New England Biolabs, Ipswich, MA). Preparation of lentiviral particles and transfection of skin fibroblasts (MOI = 2, 5, or 30) were conducted as described earlier. PYCR1 overexpression was verified through real-time PCR and Western blotting.

### Statistical analysis

Data are reported as means and standard deviations (SDs) unless indicated otherwise. The significance of pairwise comparisons was determined by Student’s t-test.

**Figure 1.**
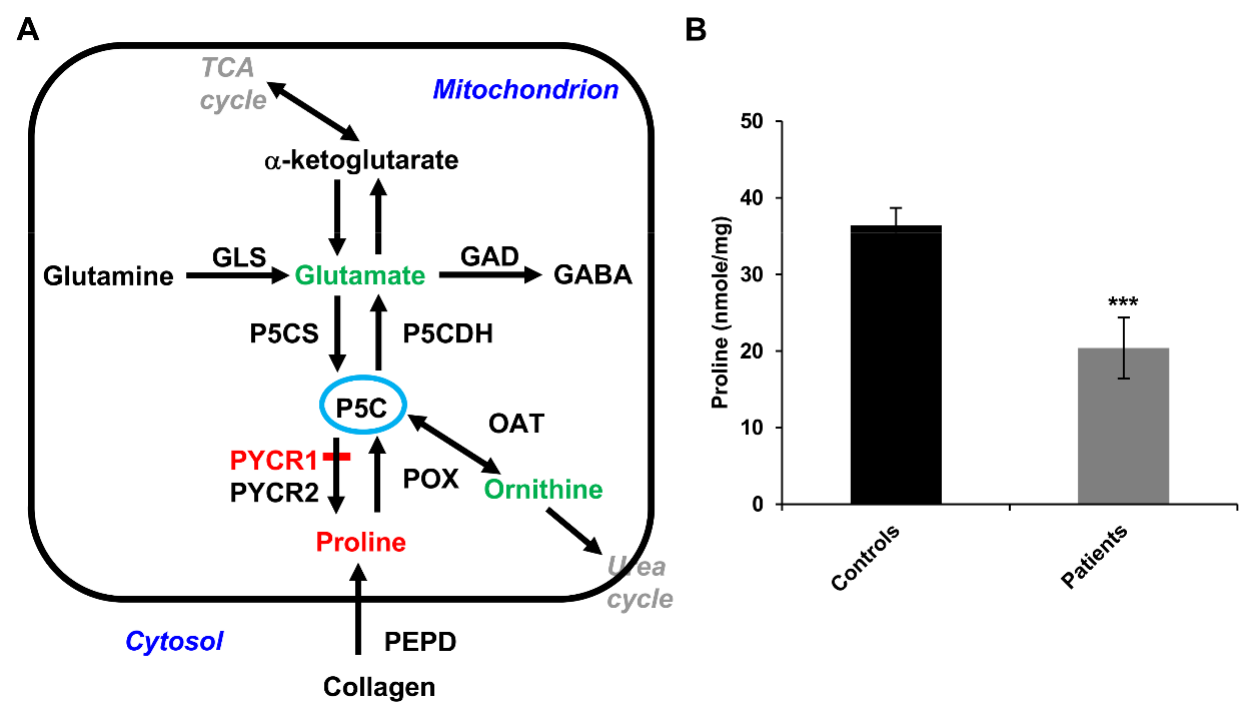
Intracellular proline levels of ARCL2B patient primary skin fibroblasts are lower than healthy controls. (A) Overview of proline biosynthesis. Proline is synthesized via glutamate and ornithine routes. The enzymes P5CS and OAT catalyze the conversion of glutamate and ornithine, respectively, to P5C which is subsequently converted to proline by PYCR1 and PYCR2. (B) Intracellular proline levels in the fibroblasts from healthy controls and patients with *PYCR1* mutations (n=4). P5C: Δ^1^-pyrroline-5-carboxylate; GLS: Glutaminase; GAD: Glutamate decarboxylase; GABA: γ- aminobutyric acid; P5CS: P5C synthase; P5CDH: P5C dehydrogenase; OAT: Ornithine aminotransferase; POX: Proline oxidase; PEPD: Proline dipeptidase; ***: *p* < 0.001

## RESULTS

### Clinical features of ARCL2B individuals with PYCR1 mutations

The clinical features of four affected individuals (P1 to P4) from three families are listed in Table 1. DNA sequencing analysis of DNA from bloods of patients and parents identified three mutations in *PYCR1 *gene. Two of the patients (P2 and P3) had a common homozygous single-base deletion (c.345delC) in exon 4, leading to a frame shift and premature termination of translation (p.P115fsX7) of PYCR1. The other two patients had compound heterozygous mutations, including the same single-base deletion (c.345delC) and the missense 743G-to-A (P1) and 559G-to-A (P4) transitions in *PYCR1*. The clinical features of patients P1, P2 and P3 have been reported in our previous papers [11, 12]. Patient P4, a 4-year-old male, was the only child of healthy parents. He was born by cesarean section at 38 weeks gestation following an uneventful pregnancy. His birth weight (2.6 kg, 10th percentile) and length (46 cm, 4th percentile) were at the lower range of norm, while his head circumference (30.5 cm) was less than the third percentile. Physical examination at birth revealed prominent forehead, wide fontanels, fine and sparse hair, blue sclera, strabismus, back placed and large helices, wrinkled and translucent skin with prominent superficial venous reticule over the trunk and root of limbs, and hypotonia. Both the karyotyping analysis and sonographies of the brain and abdominal organs at birth did not reveal any abnormalities. He had dislocation of left hip joints and bilateral inguinal hernia which were surgical corrected at the age of 8 months and 2 years, respectively. Development milestones were delayed that he began to walk at the age of 1 year and 2 months and could still spoke only few words at the age of 2 years.

**Table 1:** Clinical features manifested in four ARCL2B patients with *PYCR1* mutations.

	Family	1	2		3
	Patient ID	P1	P2	P3	P4

	Gender	Male	Male	Female	Male
	Age	6	9	8	4
PYCR1 mutations	Status	Heterozygous	Homozygous	Homozygous	Heterozygous
	Exon	4 & 6	4	4	4 & 5
	CDS	c.345delC	c.345delC	c.345delC	c.345delC
		c.743G>A			c.559G>A
	Protein	p.P115fsX7	p.P115fsX7	p.P115fsX7	p.P115fsX7
		p.G248E			p.A187T

Clinical features	Lax wrinkled skin	+	+	+	+
	Typical facial gestalt	+	+	+	+
	Hernias	+	+	­	+
	Preglaucoma	+	ND	ND	+
	Cataract	+	­	­	+
	Esotropia	+	ND	ND	+
	Aortic root dilatation Sinus of Valsalva (mm) (Range) Z-Score	+ + 23.6 (13.56-20.81) 3.47	- 19.8 (14.16-21.71) 0.97	- 19.1 (16.59-25.55) -0.86	+ 22.5 (14.05-21.54) 2.46
	Cardiovascular system	IRBBB	MR, PR	TR, MR, RAA	TR, AR
	Osteopenia	+	+	+	+
	Joint hyperlaxity	+	+	+	+
	Adducted thumb	+	+	+	+
	Hip dislocation	+	+	+	+
	Shoulder dislocation	­	+	+	-
	Abnormal brain MRI	­	­	­	­
	Mental retardation	++	++	+	+/-
	Autism	+	+	­	­
	Athetoid movements	+	++	­	­

Clinical features (neonatal)	Thin, translucent skin	+	+	+	+
	Postnatal growth delay	+	+	+	-
	Hypotonia	+	+	+	+
	Late fontanel closure	+	+	+	+
	Blue sclera	+	+	+	+
	Strabismus	+	­	­	+

IRBBB: incomplete right bundle branch block; MR: mitral regurgitation; PR: pulmonary regurgitation; TR: tricuspid regurgitation; RAA: right aortic arch pressure gradient; AR: aortic regurgitation. ND, not done; -, absent; +/-, mild; +, moderate; ++, severe.

**Table 2:** List of 54 differentially expressed gene candidates in ARCL2B patients’ primary skin fibroblasts.

Gene Symbol	Gene Description	Fold Change (Patients/ Controls)^a^	Subcellular Location	Functional Type
**Genes downregulated in patients**				
*POSTN*	Periostin	-17.22	Extracellular space	Other
*MMP1*	Matrix metallopeptidase 1	-12.22	Extracellular space	Peptidase
*PTGS2*	Prostaglandin-endoperoxide synthase 2	-9.64	Cytoplasm	Enzyme
*EFEMP1*	EGF containing fibulin like extracellular matrix protein 1	-8.69	Extracellular space	Enzyme
*IGFBP3*	Insulin like growth factor binding protein 3	-4.44	Extracellular space	Other
*JAG1*	Jagged 1	-4.37	Extracellular space	Growth factor
*MYO1D*	Myosin ID	-4.27	Cytoplasm	Enzyme
*ELN*	Elastin	-3.76	Extracellular space	Other
*C1S*	Complement C1s	-3.61	Extracellular space	Peptidase
*DCN*	Decorin	-3.45	Extracellular space	Other
*CYP1B1*	Cytochrome p450 family 1 subfamily B member 1	-3.43	Cytoplasm	Enzyme
*PTX3*	Pentraxin 3	-3.35	Extracellular space	Other
*STK32B*	Serine/threonine kinase 32B	-3.26	Other	Kinase
*C1R*	Complement C1r	-3.11	Extracellular space	Peptidase
*FBLN2*	Fibulin 2	-3.06	Extracellular space	Other
*ADAM12*	ADAM metallopeptidase domain 12	-2.96	Plasma membrane	Peptidase
*SPOCK1*	SPARC/osteonectin, cwcv and kazal like domains proteoglycan 1	-2.95	Extracellular space	Other
*CEBPD*	CCAAT/enhancer binding protein delta	-2.85	Nucleus	Transcription regulator
*COL3A1*	Collagen type III alpha 1 chain	-2.54	Extracellular space	Other
*ADAMTS5*	ADAM metallopeptidase with thrombospondin type 1 motif 5	-2.51	Extracellular space	Peptidase
*LGR4*	Leucine rich repeat containing G protein-coupled receptor 4	-2.44	Plasma membrane	Transmembrane receptor
*ARL4C*	ADP ribosylation factor like GTPase 4C	-2.43	Nucleus	Enzyme
*ACKR4*	Atypical chemokine receptor 4	-2.38	Plasma membrane	G-protein coupled receptor
*EMILIN2*	Elastin microfibril interfacer 2	-2.30	Extracellular space	Other
*JUNB*	*JUNB* proto-oncogene, AP-1 transcription factor subunit	-2.28	Nucleus	Transcription regulator
*NREP*	Neuronal regeneration related protein	-2.25	Cytoplasm	Other
*NNMT*	Nicotinamide N-methyltransferase	-2.14	Cytoplasm	Enzyme
*ALPK2*	Alpha kinase 2	-2.13	Nucleus	Kinase
*LMCD1*	LIM and cysteine rich domains 1	-2.09	Cytoplasm	Transcription regulator
*NPR3*	Natriuretic peptide receptor 3	-2.07	Plasma membrane	G-protein coupled receptor
*VEGFA*	Vascular endothelial growth factor A	-2.05	Extracellular space	Growth factor
*THBS2*	Thrombospondin 2	-2.03	Extracellular space	Other

**Genes upregulated in patients**
*HOXB7*	Homeobox B7	23.53	Nucleus	Transcription regulator
*STMN2*	Stathmin 2	10.52	Plasma membrane	Other
*TRHDE*	Thyrotropin releasing hormone degrading enzyme	7.12	Plasma membrane	Peptidase
*SPP1*	Secreted phosphoprotein 1	3.66	Extracellular space	Cytokine
*EPDR1*	Ependymin related 1	3.54	Nucleus	Other
*LBH*	Limb bud and heart development	3.04	Nucleus	Transcription regulator
*QPRT*	Quinolinate phosphoribosyltransferase	2.92	Cytoplasm	Enzyme
*CRIP2*	Cysteine rich protein 2	2.79	Nucleus	Other
*CRIP1*	Cysteine rich protein 1	2.68	Cytoplasm	Other
*MEIS1*	Meis homeobox 1	2.58	Nucleus	Transcription regulator
*PRLR*	Prolactin receptor	2.46	Plasma membrane	Transmembrane receptor
*CBR3*	Carbonyl reductase 3	2.37	Cytoplasm	Enzyme
*PMAIP1*	Phorbol-12-myristate-13-acetate-induced protein 1	2.33	Cytoplasm	Other
*PTN*	Pleiotrophin	2.28	Extracellular space	Growth factor
*ATF5*	Activating transcription factor 5	2.20	Nucleus	Transcription regulator
*CSPG4*	Chondroitin sulfate proteoglycan 4	2.20	Plasma membrane	Other
*SH2D5*	SH2 domain containing 5	2.19	Plasma membrane	Other
*AMIGO2*	Adhesion molecule with Ig like domain 2	2.18	Plasma membrane	Other
*CNIH3*	Cornichon family ampa receptor auxiliary protein 3	2.17	Plasma membrane	Transporter
*HIST1H4H*	Histone cluster 1 H4 family member h	2.15	Nucleus	Other
*ANKRD30BL*	Ankyrin repeat domain 30B like	2.11	Other	Other
*ADGRE5*	Adhesion g protein-coupled receptor E5	2.08	Plasma membrane	G-protein coupled receptor

a TPM ≥ 10 in patients or controls and fold change ≥ 2

At the time of our assessment at 4 years old, patient P4 has achieved weight (19.6 kg) and height (110.5 cm) catch-up. Clinical assessment demonstrated features of prominent forehead, large helices, protruding ears, hypermobility of small joints of the hands and feet, and wrinkled and inelastic skin with prominent veins over the dorsum of the hands and feet. The ophthalmic examination revealed preglaucoma, estropia, amblyopia, astigmatism and mild vitreous opacities. Brain MRI revealed no abnormality, while echocardiographic evaluation revealed dilated aortic root with Z-score of 2.46 [18] and skeletal radiography revealed scoliosis. Psychological assessment revealed poor attention and mild intellectual disability with full scale intellectual quotient of 69 at age of 4 years. He received cataract surgery in the right eye at age of 5 years and in the left eye at 8 years.

PYCR1 deficiency leads to a premature aging phenotype in multiple systems, including vision (preglaucoma and cataract), cardiovascular system (aortic root dilatation and valve regurgitation), bone (osteopenia, joint hyperlaxity, and hip dislocation), central nervous system (CNS; mental retardation, autism, and athetoid movements), typical triangular facial gestalt, and progeroid cutaneous manifestations [5, 6, 10-12]. Notably, all four patients had cardiovascular diseases, which has not been reported before.

### PYCR1 mutations associated with reduced proline level and PYCR1 expression in primary skin fibroblasts

**Figure 2.**
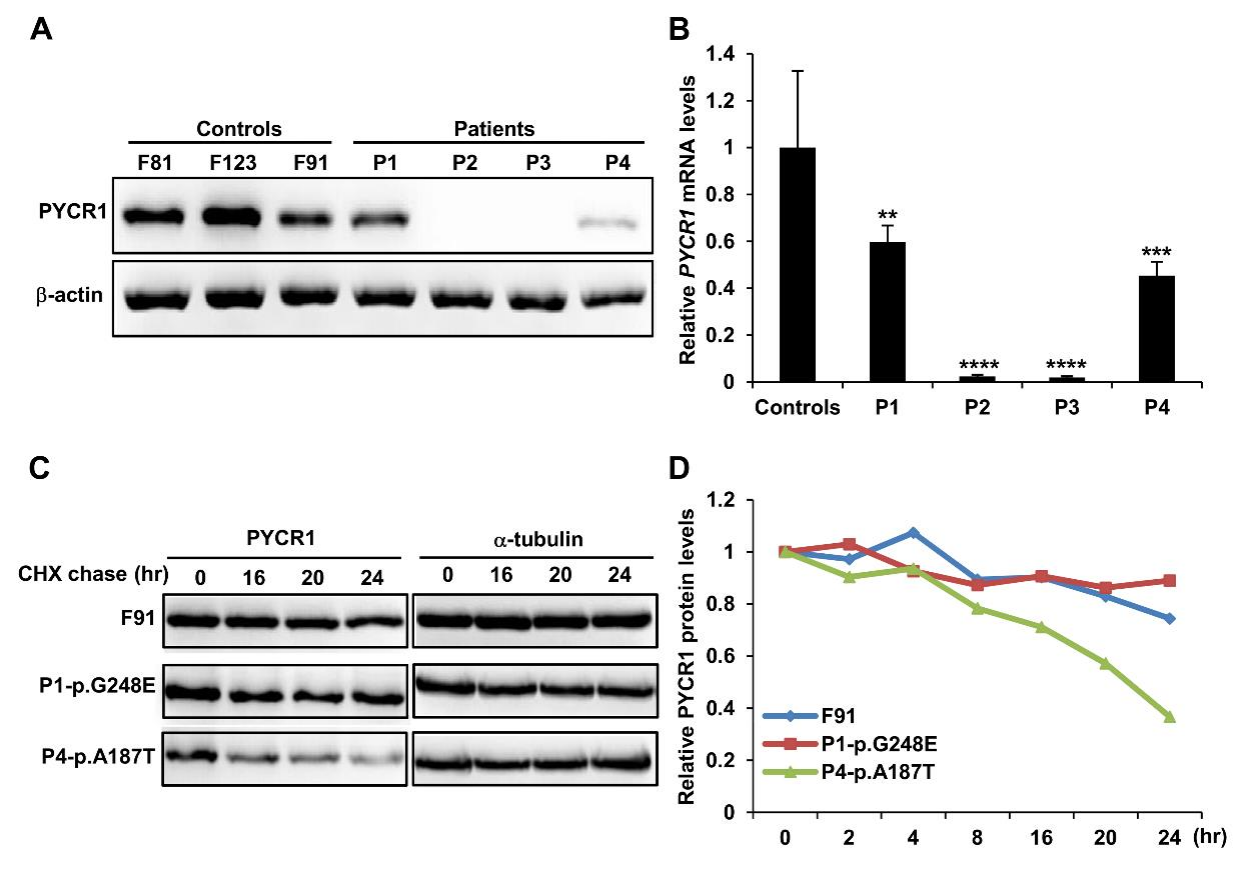
*PYCR1* mutations affected its mRNA and protein levels in patients’ primary skin fibroblasts. (A) Immunoblot showing the truncated PYCR1 protein p.P115fsX7 is undetectable in fibroblasts from patients (P2 and P3) with homozygous c.345delC mutation, whereas the mutant proteins p.G248E (P1) and p.A187T (P4) are detected in samples with heterozygous mutations. (B) *PYCR1* levels relative to *GAPDH* in four each of healthy control and patients were determined by SYBR Green based qRT-PCR. Data is expressed as mean ± SD from three independent experiments. ** *p* < 0.01, *** *p* < 0.001, and **** *p* < 0.0001. (C) Different single amino acid substitutions in P1 and P4 impeded the PYCR1 half-life as determined by cycloheximide (CHX) chase assay. Cells were harvested and lysed at 0, 4, 8, 16, and 24 h after treatment in medium containing 20 µg/ml CHX. (D) Results in (C) were quantified using the IMAGE J software. α-tubulin was used to normalize the PYCR1 protein levels. The relative levels at 0 hr were defined as 1 for each sample.

PYCR1 catalyzes the final step in proline synthesis, in which pyrroline-5-carboxylate is converted to proline in the mitochondria; thus, PYCR1 defects may affect proline synthesis (Fig. 1A). Examination of primary skin fibroblasts revealed that patients with *PYCR1* mutations had lower intracellular proline levels (Fig. 1B). The truncated PYCR1 protein p.P115fsX7 was not detectable in fibroblasts from patients P2 and P3 with homozygous c.345delC mutations as revealed by Western blotting, whereas different levels of p.G248E and p.A187T mutant proteins, but not the truncated form, were found in fibroblasts from patients P1 and P4 with heterozygous *PYCR1* mutations, respectively (Fig. 2A and 2B). The observed differences between patients with homozygous and heterozygous *PYCR1* mutations indicated that the premature termination codons resulting from the c.345delC mutation might have caused the downregulation of *PYCR1* mRNA due to nonsense-mediated mRNA decay, and that the amino acid substitutions might have affected protein stability. Furthermore, pulse-chase analysis indicated that the PYCR1 p.G248E mutant protein showed a slower degradation rate than the p.A187T mutant protein (Fig. 2C and 2D). In addition, patient with the p.G248E mutant protein had more severe neurological and orthopedic manifestations, suggesting that p.G248E affects protein function.

### Expression of genes encoding ECM components was reduced in fibroblasts with PYCR1 mutations

**Figure 3.**
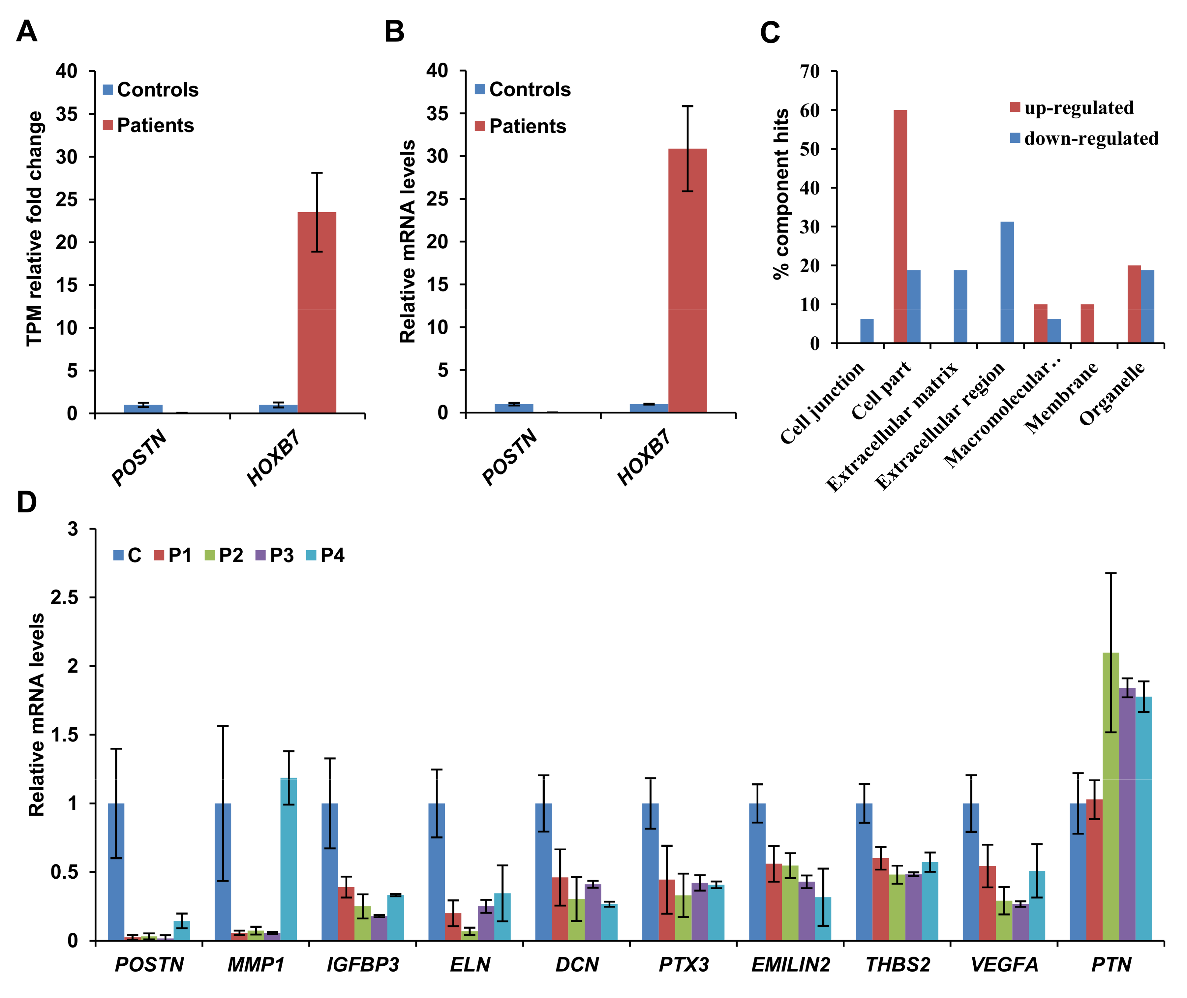
qRT-PCR validated the expression changes of *PYCR1* affected extracellular space protein genes. (A) Relative change of the most up- and downregulated expressed genes as determined by RNA-Seq analysis. (B) qRT-PCR validation of the two candidates in (A). (C) Gene ontology (GO) analysis shows enrichment of genes related to extracellular marix/region components are downregulated in patients’ fibroblasts. (D) The expression levels of ten extracellular space protein genes (fold-change > 2, TPM > 10) in the skin fibroblasts of all four patients and three different healthy donors were quantified by qRT-PCR. Data is expressed as mean ± SD from three independent experiments. * *p* < 0.05, ** *p* < 0.01.

RNA-Seq transcriptome analysis of two patients (P1 and P3) and two controls detected approximately 60.8 to 75.9 million read pairs per sample, which were unambiguously mapped to 21,141 reference gene sequences (NCBI Build 37/hg19 database; total 23,769 human genes). Among the putative differentially expressed genes with at least 2-fold change and a minimum of 10 TPM in patients or controls, sequence annotations indicated the proteins of 17 (53.1%) of the genes downregulated and 2 (9.0%) of the the genes upregulated in patients are localized in the extracellular space. Expression changes of the most downregulated gene periostin *(POSTN) *and the most upregulated gene homeobox B7 (*HOXB7*) (Table 2 and Fig. 3A) in fibroblasts from patients were confirmed through SYBR Green-based qRT-PCR (Fig. 3B).

Gene Ontology analysis of the differentially expressed candidates for cellular component distributions using the *PANTHER Classification System* indicated ECM and extracellular region proteins were enriched in patients with *PYCR1* mutations (www.pantherdb.org/). A biased distribution of essentially downregulated candidates of ECM and extracellular protein genes was found (Fig. 3C). These included genes encoding the ECM/extracellular region proteins POSTN, metallopeptidase 1 (MMP1), insulin-like growth factor-binding protein 3 (IGFBP3), ELN, complement C1s (C1S), ADAM metallopeptidase with thrombospondin type 1 motif 5 (ADAMTS5), and vascular endothelial growth factor A (VEGFA).

Among the aforementioned proteins, the downregulated ECM components, elastin (ELN) and decorin (DCN), are associated with different phenotypes of aging, such as cutis laxa and joint and dermal manifestations [3, 19]. In addition to these two proteins, the mRNA levels of seven other downregulated extracellular space proteins, POSTN, MMP1, IGFBP3, pentraxin 3 (PTX3), EMILIN2, thrombospondin 2 (THBS2), and vascular endothelial growth factor A (VEGFA), and the upregulated ECM protein pleiotrophin (PTN) were quantified using qRT-PCR in a different set of controls and skin fibroblasts from all four patients (Fig. 3D). Of the 10 examined genes, *MMP1* was only downregulated in patients P1, P2, and P3, but not in P4, and *PTN* did not show significant upregulation in patient P1. Hence, *MMP1* and *PTN* were excluded for further analysis.

### Association of diseases and functions revealed by Ingenuity Pathway Analysis

**Figure 4.**
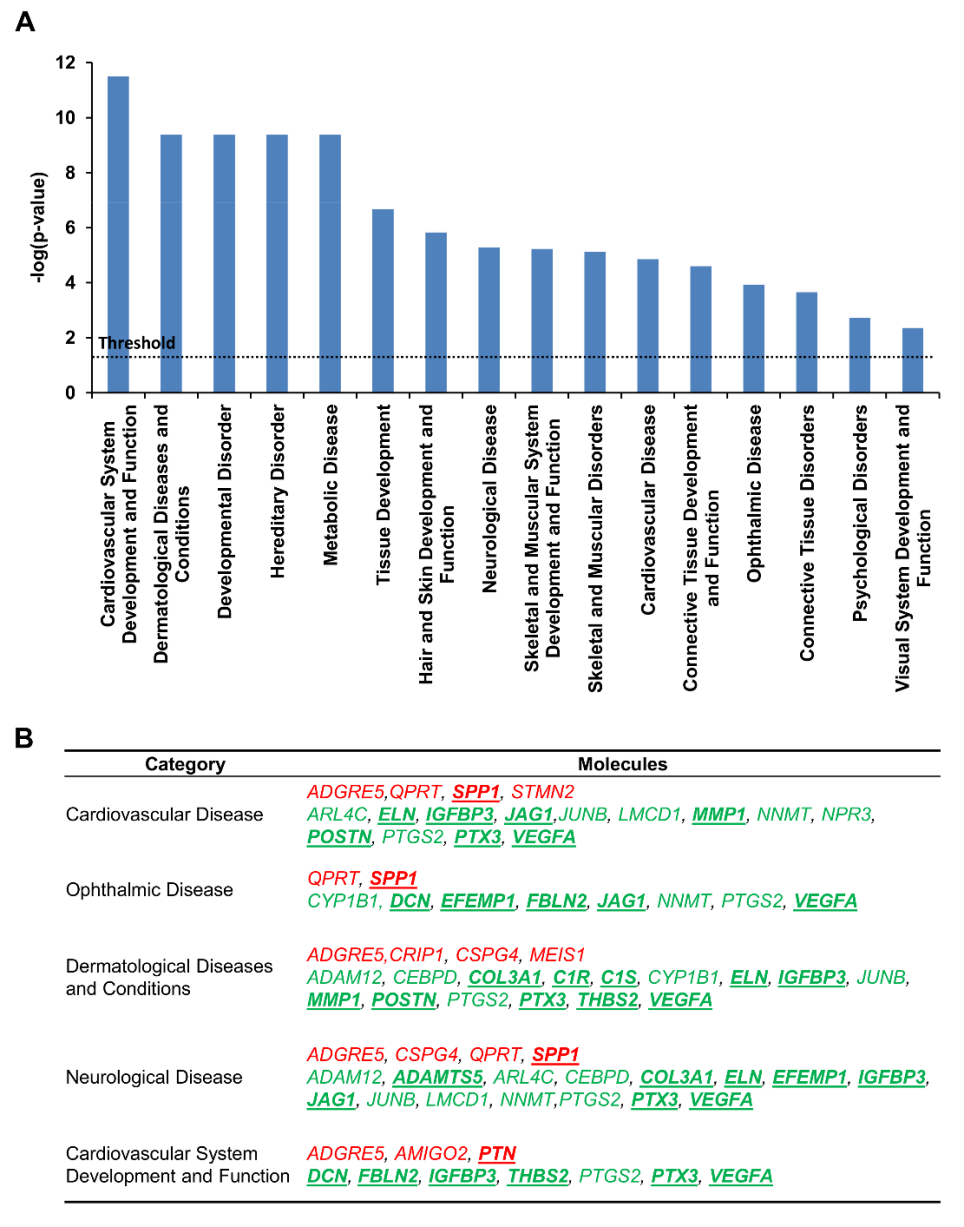
IPA analysis indicated differentially expressed gene candidates are associated with cardiovascular, ophthalmic, and dermatological diseases. Genes with fold change > 2 and TPM > 10 were analyzed. (A) Genes implicated in the principal biological function categories related to the catalogues “Diseases and Disorders” and “Physiological System Development and Function”. (B) List of the genes implicated in top five catalogues. The input putatively up- and downregulated genes are indicated by red and green texts, respectively. The molecules located in extracellular space are bold and underlined.

To explore the diseases and functions that may be related to *PYCR1* mutations, 54 differentially expressed gene candidates with TPM > 10 and with fold changes ≥ 2 were analyzed using the Ingenuity Pathway Analysis (IPA) software. With the pertinent parameter settings, IPA of “Diseases and disorders” and “Physiological system development and function” revealed these genes in 16 modules (*p* < 0.05) (Fig. 4A). The top five potentially affected modules were related to the cardiovascular system, ophthalmic disease, dermatological diseases, and neurological disease, and each contained at least five downregulated extracellular space genes (Fig. 4B). Among the differentially expressed genes, the mRNA levels of *DCN*, *EMILIN2*, *IGFBP3*, *MMP1*, *PTX3*, *THBS2*, and *VEGFA* was quantified using qRT-PCR (Fig. 3D).

### Perturbation of PYCR1 affected ECM gene expression

**Figure 5.**
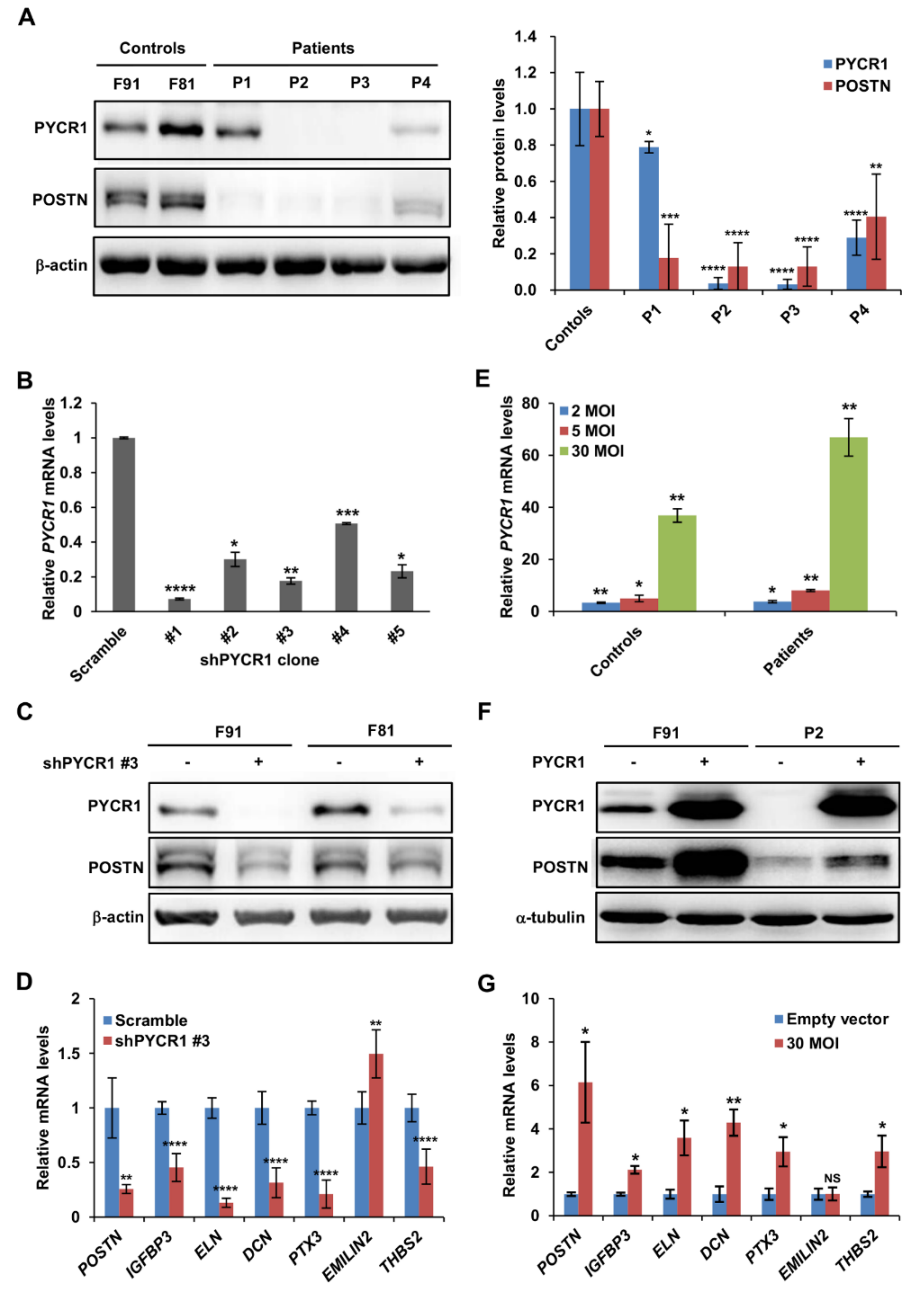
Perturbations of PYCR1 expression modulate the expression of extracellular space genes in skin fibroblast. (A) Western blot (*left*) and quantification (*right*) of PYCR1 and POSTN protein levels in primary skin fibroblasts from controls and patients. Data was expressed as means ± SD from four biological replicates. (B) Evaluation of knockdown efficiencies of pLKO.1 lentiviruses expressing five independent *PYCR1* shRNAs in primary skin fibroblasts. At 48 h post-transduction, skin fibroblasts were collected for RNA isolation and qRT-PCR. (C) At 72 h post-transduction, proteins were isolated from fibroblasts and analyzed by Western blot. Scramble shRNA was used as negative control. (D) Knockdown of *PYCR1* (shPYCR1 #3, 2MOI) in skin fibroblast modulates the expression of extracellular space genes. (E) Evaluation of the overexpression of *PYCR1* in human fibroblasts infected with 2, 5, and 30 MOI of recombinant *PYCR1* lentiviruses relative to the empty vector control. (F) Western blot analyses revealed that PYCR1 overexpression (30 MOI) induce POSTN expression in skin fibroblast cells. (G) *PYCR1* overexpression in patient’s skin fibroblasts (P2) modulates expression of extracellular space genes. Data was expressed as means ± SD from three biological replicates. NS: not significant, * *p* < 0.05, ** *p* < 0.01, *** *p* < 0.001, and **** *p* < 0.0001.

RNA-Seq analysis revealed the expression of several ECM genes and genes of other categories were altered in the primary cultures of fibroblasts from ARCL2B patients. The most downregulated gene in fibroblasts from patients in RNA-Seq analysis was *POSTN*. The expression of the POSTN protein was also decreased in fibroblasts from the four patients (Fig. 5A). The correlation between the *PYCR1* mutation and ECM gene expression was further evaluated by perturbing the PYCR1 expression in fibroblasts from controls and patients.

Modulation of PYCR1 expression via shRNA knockdown or overexpression affected the expression of almost all analyzed extracellular space genes. Transduction of skin fibroblasts with five *PYCR1-*specific shRNAs reduced *PYCR1* expression from 50% to 93% (Fig. 5B) and the expression of *POSTN*, the most downregulated gene in ARCL2B patients as detected by RNA-Seq, from 0% to 75% (Supplementary Fig. 1A). The data indicated that *POSTN* expression in skin fibroblasts was correlated with the *PYCR1* expression level (R^2 ^= 0.9143), and that shPYCR1 #1 had the highest knockdown efficiency in *PYCR1* and *POSTN* (93% and 75%, respectively). However, knockdown with shPYCR1 #1 also partly affected the expression of *PYCR2* (Supplementary Fig. 2A), most likely due to the high homology of target sequences in *PYCR1* and *PYCR2* mRNAs [20] (Supplementary Fig. 2B). Thus, instead of shPYCR1 #1, shPYCR1 #3 was chosen for further analysis because it had minor influence on PYCR2 expression. Western blot analysis also showed the downregulation of POSTN expression (Fig. 5C). Figure 5D shows the qRT-PCR expression profiling results, which indicated the reduced expression of extracellular space genes, including *POSTN*, *IGFBP3*, *ELN*, *DCN*, *PTX3*, and *THBS2*, but not *EMILIN2*, in *PYCR1* knocked-down cells. In contrast to *PYCR1* knockdown, *PYCR1* overexpression in fibroblasts from patients not only restored the intracellular proline level (Supplementary Fig. 3) to a higher level but also increased the mRNA and protein expression levels of POSTN and most of the other extracellular space genes (Fig. 5F and 5G). These results indicate that PYCR1 modulates the expression of extracellular space genes, except for *EMILIN2*.

## DISCUSSION

In this study, we provided the first characterization of the transcriptome for *PYCR1* mutations causing the neurocutaneous syndrome ARCL2B. This study also revealed previously unknown associations between the *PYCR1* mutation and molecular changes in patients with ARCL2B. In our patients, PYCR1 deficiency certainly led to a premature aging phenotype, but this is the first report of heart valves disease and preglaucoma in such patients. No effective therapeutic strategies are yet available for ARCL2B, and the underlying mechanisms involved in disease-related changes require further elucidation.

Diverse *PYCR1* mutations led to different half-lives of the resulting PYCR1 proteins. The PYCR1 mutant protein with the G248E substitution showed slower degradation than that with the A187T substitution but resulted in with more severe neurological and orthopedic manifestations, thereby suggesting that p.G248E is null at the functional level [11]. Because PYCR1 catalyzes the conversion of P5C to proline, *PYCR1* mutations are expected to lead to decreased intracellular proline levels. To confirm this, we also showed that PYCR1 overexpression increased intracellular proline levels. Hence, PYCR1 modulates proline levels in skin fibroblasts. No significant difference was observed in the intracellular proline level between patients with homozygous frameshift (P2 and P3) and compound heterozygous frameshift and missense mutations (P1 and P4). These patients only showed the truncated protein p.P115fsX7 and the truncated plus mutant protein with the G248E or A187T substitution. The results differed from those of Reversade *et al*. [5], probably due to differences in culture conditions and the measurement method. Proline and its derivative, hydroxyproline, play an essential role in the formation of collagen and elastin, which are major ECM components in many organs, such as the connective tissue. Decreases in proline levels may affect collagen and elastin synthesis in connective tissues, leading to cutis laxa [2].

Transcriptome analysis of the fibroblasts from patients’ with *PYCR1* mutations and controls revealed that many of the differentially expressed genes coded for proteins localized in the extracellular space. Genetic mutations in these proteins are associated with different phenotypes of aging. Many ECM components are found to be decreased in patients with cutaneous syndromes because alterations of ECM composition may lead to abnormal elastic fibers, thus causing cutis laxa [3]. In addition to providing structural and biochemical support to the surrounding cells, ECM also offers a dynamic and complex environment for interactions with other ECM components. Cell-matrix interactions not only mediate adhesion but also transduce signals to modulate cell survival, proliferation, differentiation, phenotype, and behavior [21]. Therefore, alterations of ECM composition during development affect cell behaviors, leading to ECM dysregulation and disease progression [22]. Furthermore, in the present study, IPA revealed that the affected genes associated with cardiovascular disease as one of the main aging-associated diseases; these genes included the genes for many extracellular space protein (*DCN*, *ELN*, *IGFBP3, POSTN*, *PTX3*, *THBS1*, and *THBS2*), which were downregulated in fibroblasts from ARCL2B patients. *POSTN*, the most downregulated gene in affected individuals, is a heterofunctional regulator of cardiac development and disease [23, 24]. Our study demonstrated for the first time that many extracellular space genes (*POSTN*, *IGFBP3*, *ELN*, *DCN*, *PTX3*, and *THBS2*) are affected by PYCR1. This study also provided a comprehensive linkage of extracellular space genes for the neurocutaneous syndrome caused by *PYCR1 *mutations.

We demonstrated that *POSTN* expression in skin fibroblasts is correlated with the *PYCR1* expression level and is significantly decreased in adult skin fibroblasts (Supplementary Fig. 1). POSTN comprises an amino-terminal EMI domain, four repeated domains related to those found in fasciclin-1 (fasciclin-1-like repeats), and a carboxyl-terminal domain. It belongs to the family of fasciclins based on its homology to fasciclin 1 (FAS1), an insect cell adhesion protein involved in CNS development. POSTN is expressed not only during embryogenesis or pathogenesis [25, 26] (including neoplasia, tissue repair, and cardiac injury) but also in various organs, particularly fibroblast-rich connective tissues such as the skin or breast [27]. POSTN has pivotal functions in heart valvulogenesis [28-30], trabecular meshwork development [31], skin wound healing [32], brain and CNS development [33, 34], and skin aging [35]. However, the role of POSTN in neurocutaneous syndrome has never been described. Studies have shown that *CYP1B1* is a causative gene in primary congenital glaucoma [36, 37], and that the phenotype of *Cyp1b1*^-/-^ mouse model resembled glaucomatous human eyes [31]. Using the *Cyp1b1*^-/-^ mouse model, a study found that CYP1B1 deficiency increased oxidative stress and decreased POSTN production, leading to trabecular meshwork tissue dysgenesis and dysfunction [31]. We also found that PYCR1 modulated *CYP1B1* expression in skin fibroblasts (Supplementary Fig. 4). Taken together, POSTN may play a crucial role in skin aging, heart valve disease, and preglaucoma.

PYCR1 deficiency also leads to irregular, fragmented, and reduced elastic fibers [5, 6]. Our study found that *ELN* expression might be regulated by PYCR1. ELN is a major component of ECM; thus, its loss will affect ECM assembly. Expression of microfibril and elastic fiber-associated molecules such as *DCN*, *EMILIN2* (Fig. 3D), *TGFBI*, FBLN2, and FBLN3 (*EFEMP1*) (Supplementary Fig. 5) were also reduced in fibroblasts from ARCL2B patients. These molecules provide structural support to organs and tissues such as the heart, skin, lungs, ligaments, and blood vessels. These findings suggested that PYCR1 deficiency caused the loose arrangement of elastic fibers and the presence of fine and short fragmented microfibrils.

CRIP2, which plays a crucial role in atrioventricular valve development [38] and coronary smooth muscle differentiation [39], was one of the most upregulated genes in affected individuals, but no significant change in *PYCR1* knockdown or overexpression was found (Supplementary Fig. 6). The result suggests that the upregulation of CRIP2 in skin fibroblasts from affected individuals is not modulated by PYCR1 and may be a dosage compensation effect. Furthermore, our data also indicate that *PTN* is not modulated by PYCR1. PTN is a secreted cell signaling growth factor and development-regulated cytokine, also known as osteoblast-specific factor 1 (OSF-1); it has multiple effects on cell proliferation, differentiation, and angiogenesis. It becomes re-activated in response to injury. PTN had been reported to have roles in bone [40] and heart injury repair [41] and as a neuromodulator [42]. A previous study found that PTN was upregulated in the heart of *Postn*^-/-^ mice, and that POSTN suppressed PTN expression [43]. This suggests that our findings may be extendable to the human disease.

There are two paralogs of PYCR1 in humans - the highly similar PYCR2 (84% homology) and the more distantly related PYCRL (45% homology to the other two forms). Both PYCR1 and PYCR2 are thought to localize to the mitochondria and primarily catalyze the conversion of glutamate to proline, and PYCRL is a cytoplasmic enzyme [44]. A study indicated no significant changes in *PYCR2* expression in fibroblasts from individuals with *PYCR1* mutations [5]. We did not observe the dosage compensation of *PYCR2* and *PYCRL* in affected individuals (Supplementary Fig. 7). Recently, Nakayama *et al*. showed that *PYCR2* mutations caused microcephaly and hypomyelination in humans [45]. The different phenotypes caused by the *PYCR1* and *PYCR2* variants and their different expression levels in various tissues suggest that each of these isozymes has a unique role in the human body.

In conclusion, we found that *PYCR1* mutations significantly reduced the expression of many extracellular space genes (*POSTN, IGFBP3*, *ELN*, *DCN, PTX3,* and *THBS2*), and that PYCR1 may modulate the expression of these extracellular space genes in skin fibroblasts. *ELN* mutations resulting in autosomal-dominant cutis laxa are known to be associated with different phenotypes of aging (cutis laxa and cardiovascular and musculoskeletal abnormalities), and the *DCN* mutation leads to abnormal collagen fibril morphology and skin fragility [3, 46, 47]. Most importantly, we found a novel candidate POSTN, which was the major downregulated gene in affected individuals and the expression of which in skin fibroblasts cells was correlated with PYCR1 expression. POSTN has never been described in PYCR1-related ARCL, and our results indicate that POSTN may play an important role in skin aging, heart valves disease, and preglaucoma.

## Supplementary data

Supplementary data is available online at www.aginganddisease.org/EN/10.14336/AD.2018.0222
