# Supplementary material for: A Transcriptome Study of Progeroid Neurocutaneous Syndrome Reveals POSTN As a New Element in Proline Metabolic Disorder
Source: Aging Dis. 2018 Dec 4;9(6):1043–57. doi: 10.14336/AD.2018.0222 (PMC6284769; doi:10.14336/AD.2018.0222)
Supplement: Supplementary file 1 — Supplementary data is available online at www.aginganddisease.org/EN/10.14336/AD.2018.0222 [file AD-9-6-1043-s.pdf]

SUPPLEMENTARY DATA

A Transcriptome Study of Progeroid Neurocutaneous Syndrome Reveals POSTN As a New Element in Proline Metabolic Disorder

Yu-Wen Huang[1,2], Ming-Fu Chiang[3,4,5], Che-Sheng Ho[6], Pi-Lien Hung[7], Mei-Hsin Hsu[7], Tsung-Han Lee[2], Lichieh Julie Chu[8], Hsuan Liu[8,9], Petrus Tang[10], Wailap Victor Ng[1,11,12,*], Dar-Shong Lin[2,6,13,*]

[1]Institute of Biotechnology in Medicine and Department of Biotechnology and Laboratory Science in Medicine, National Yang Ming University, Taipei, Taiwan. [2]Department of Medical Research, Mackay Memorial Hospital, Taipei, Taiwan. [3]Department of Neurosurgery, Mackay Memorial Hospital, Taipei, Taiwan. [4]Mackay Junior College of Medicine, Nursing and Management, Taipei, Taiwan. [5]Graduate Institute of Injury Prevention and Control, Taipei Medical University, Taipei, Taiwan. [6]Department of Pediatrics, Mackay Memorial Hospital, Taipei, Taiwan. [7]Department of Pediatric Neurology, Kaohsiung Chang Gung Memorial Hospital, and Chang Gung University College of Medicine, Kaohsiung, Taiwan. [8]Molecular Medicine Research Center, Chang Gung University, Taoyuan, Taiwan. [9]Department of Cell and Molecular Biology, College of Medicine, Chang Gung University, Taoyuan, Taiwan. [10]Molecular Regulation and Bioinformatics Laboratory and Department of Parasitology, Chang Gung University, Taoyuan, Taiwan. [11]Institute of Biomedical Informatics and Center for Systems and Synthetic Biology, National Yang Ming University, Taipei, Taiwan. [12]Department of Biochemistry, Kaohsiung Medical University, Kaohsiung, Taiwan. [13]Department of Medicine, Mackay Medical College, New Taipei, Taiwan

© 2018. Huang YW et al. Published online at http://www.aginganddisease.org/EN/10.14336/AD.2018.0222

SUPPLEMENTARY DATA

Supplementary Table 1. Sequences of the human cDNA primers used for qRT-PCR and PCR.

Accession number	Gene name	Primer name	Primer Sequence 5' to 3'	Product size (bp)
NM_001282280	*PYCR1*	PYCR1-2F<br>PYCR1-3R	ATGAGCGTGGGCTTCATC<br>GGGAGCTAGCCATTATCTTGTG	103
NM_001135935	*POSTN*	POSTN-9F<br>POSTN-10R	CAGCAAACCACCTTCACGGATC<br>TTAAGGAGGCGCTGATCCATGC	140
NM_004502	*HOXB7*	HOXB7-1F<br>HOXB7-2R	ATCTACCCCTGGATGCGAAGCT<br>GCGTCAGGTAGCGATTGTAGTG	115
NM_001312	*CRIP2*	CRIP2-6F<br>CRIP2-7R	TGACGTCTCTGGGCAAGGATTG<br>CCGAAGAGGATTCCATAGCAGG	136
NM_002421	*MMP1*	MMP1-2F<br>MMP1-3R	ATGAAGCAGCCCAGATGTGGAG<br>TGGTCCACATCTGCTCTTGGCA	137
NM_001013398	*IGFBP3*	IGFBP3-2F<br>IGFBP3-3R	CGCTACAAAGTTGACTACGAGTC<br>GTCTTCCATTTCTCTACGGCAGG	105
NM_000501	*ELN*	ELN-17F<br>ELN-18R	GGTTGTGTCACCAGAAGCAGCT<br>CCGTAAGTAGGAATGCCTCCAAC	96
NM_001920	*DCN*	DCN-5F<br>DCN-6R	GCTCTCCTACATCCGCATTGCT<br>GTCCTTTCAGGCTAGCTGCATC	128
NM_002852	*PTX3*	PTX3-1F<br>PTX3-2R	CGAAATAGACAATGGACTCCATCC<br>CTCATCTGCGAGTTCTCCAGCA	111
NM_032048	*EMILIN2*	EMILIN2-2F<br>EMILIN2-3R	CAACTGTGCCTGGAACCAGATG<br>AAAGCCAGGACAGCACCTCCAT	124
NM_003247	*THBS2*	THBS2-9F<br>THBS2-10R	CAGTCTGAGCAAGTGTGACACC<br>TTGCAGAGACGGATGCGTGTGA	120
NM_001025366	*VEGFA*	VEGFA-1F<br>VEGFA-2R	TTGCCTTGCTGCTCTACCTCCA<br>GATGGCAGTAGCTGCGCTGATA	126
NM_002825	*PTN*	PTN-3F<br>PTN-4R	TGGAGCTGAGTGCAAGCAAACC<br>CTGGAACTGGTATTTGCACTCCG	97
NM_000104	*CYP1B1*	CYP1B1-2F<br>CYP1B1-3R	GCCACTATCACTGACATCTTCGG<br>CACGACCTGATCCAATTCTGCC	129
NM_000358	*TGFBI*	TGFBI-8F<br>TGFBI-9R	GGACATGCTCACTATCAACGGG<br>CTGTGGACACATCAGACTCTGC	149
NM_000660	*TGF-β1*	TGFB1-2F<br>TGFB1-3R	TACCTGAACCCGTGTTGCTCTC<br>GTTGCTGAGGTATCGCCAGGAA	122
NM_013328	*PYCR2*	PYCR2-2F<br>PYCR2-3R	CGGCTCACAAGATAATAGCC<br>TTCACCGTCTCCTTGTTGC	106
NM_023078	*PYCRL*	PYCRL-2F<br>PYCRL-3R	GTGGAAGCTCAGCACATACTGG<br>CTTGGTGGCAAAGATGACGAGC	135
NM_002046	*GAPDH*	GAPDH-7F<br>GAPDH-8R	TGCACCACCAACTGCTTAGC<br>GGCATGGACTGTGGTCATGAG	87
NM_001282280	*PYCR1*	PYCR1-EcoRI<br>PYCR1-*Bam*HI	ATCGAGAATTCACAGCATGAGCGTGGGCTT<br>ATCGGGATCCTGGCAGGATGGTGGTCA	1014

© 2018. Huang YW et al. Published online at http://www.aginganddisease.org/EN/10.14336/AD.2018.0222

SUPPLEMENTARY DATA

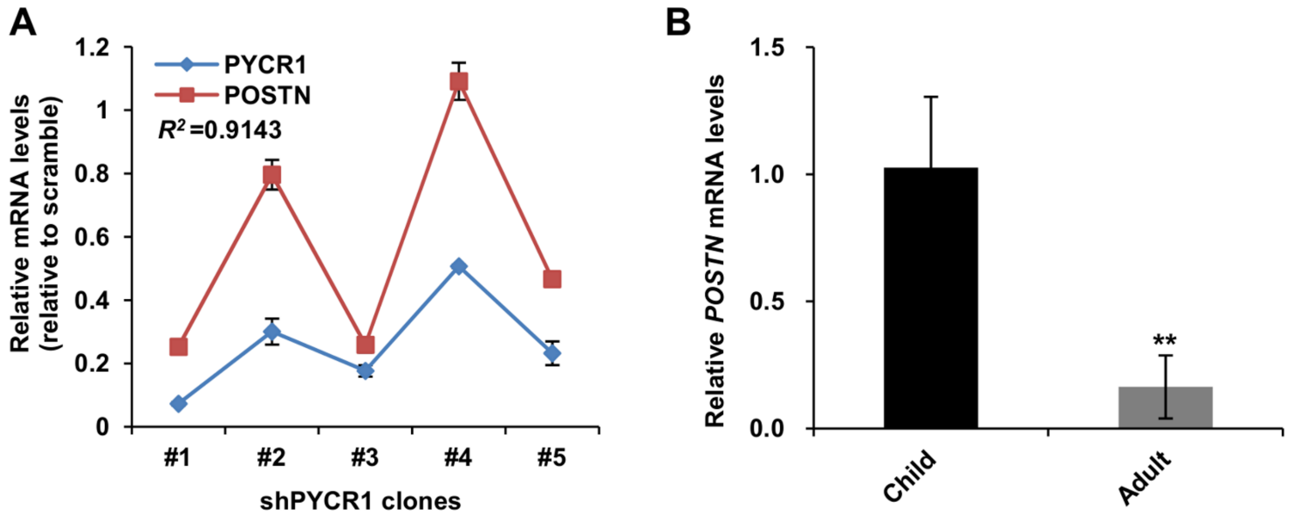

Supplementary Figure 1. *POSTN* expression level is correlated with *PYCR1* expression level and reduced in aged donors' skin fibroblasts. (**A**) *PYCR1* and *POSTN* expression changes were examined in cells infected with five independent *PYCR1* shRNAs clones. (**B**) qRT-PCR analysis of *POSTN* expression in skin fibroblasts from children (7.6 ± 5 years) and adults (40.6 ± 6 years) (n=3). Data was expressed as means ± SD of three independent experiments. ** $p < 0.01$.

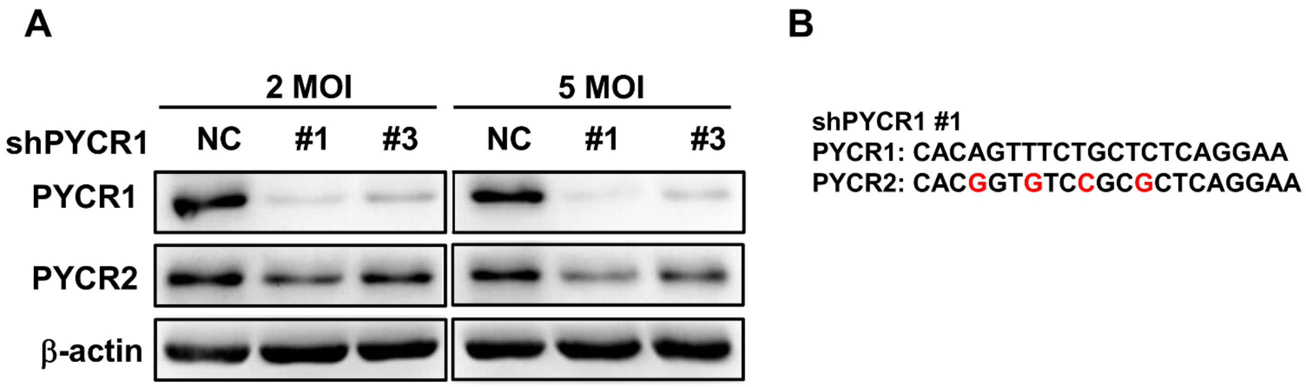

Supplementary Figure 2. shPYCR1 knockdown of PYCR1 in control skin fibroblasts partly reduced the expression of PYCR2. (**A**) Western blot analysis of the lysates from control primary skin fibroblasts infected with shPYCR1 #1 and #3 (2 MOI and 5 MOI). (**B**) Alignment of the shPYCR1 #1 targeted *PYCR1* sequence and a potential *PYCR2* cross-reacting site. *PYCR2* mismatched residues are shown in red.

© 2018. Huang YW et al. Published online at http://www.aginganddisease.org/EN/10.14336/AD.2018.0222

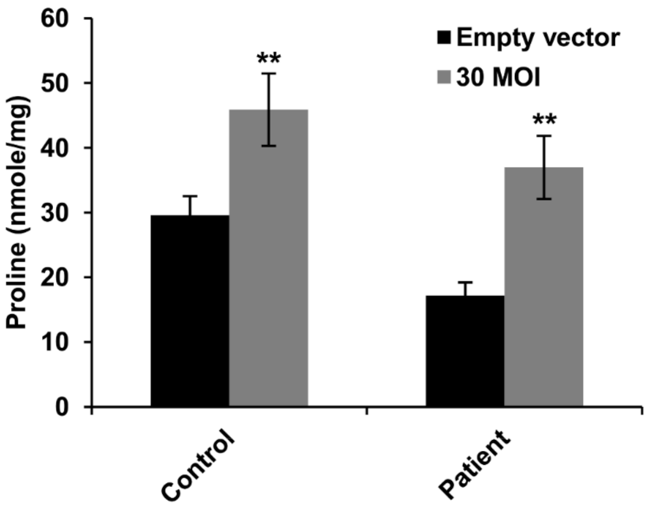

Supplementary Figure 3. Intracellular proline level is increased in PYCR1 overexpressed skin fibroblasts. Control and patient primary skin fibroblasts with PYCR1 overexpression (30 MOI) were lysed and analyzed for intracellular proline levels in four independent experiments. ** $p < 0.01$.

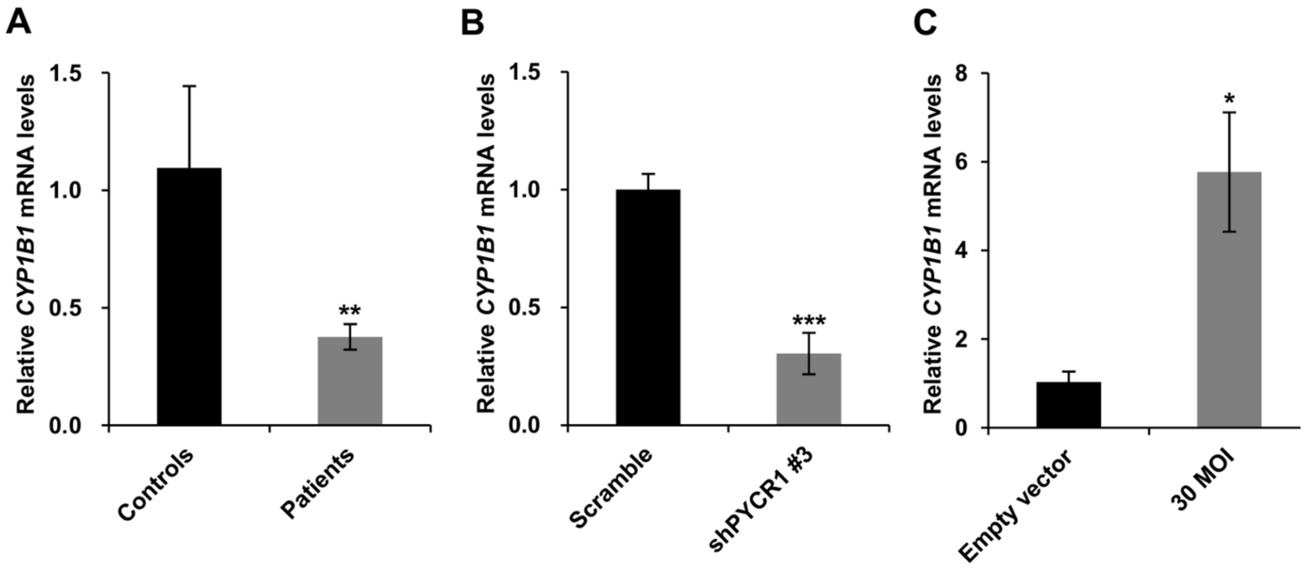

Supplementary Figure 4. *PYCR1* modulates the expression of *CYP1B1* in skin fibroblasts. (**A**) qRT-PCR analysis of *CYP1B1* gene expression in primary skin fibroblasts from controls (n=3) and patients (n=4). Data was expressed as means ± SD of three independent experiments. (**B**) *CYP1B1* expression in *PYCR1* knockdown (shPYCR1 #3; 2 MOI) skin fibroblasts of two independent experiments. (**C**) *CYP1B1* expression in *PYCR1* overexpressed (30 MOI) skin fibroblasts of three independent experiments. ** $p < 0.01$ and *** $p < 0.001$.

© 2018. Huang YW et al. Published online at http://www.aginganddisease.org/EN/10.14336/AD.2018.0222

SUPPLEMENTARY DATA

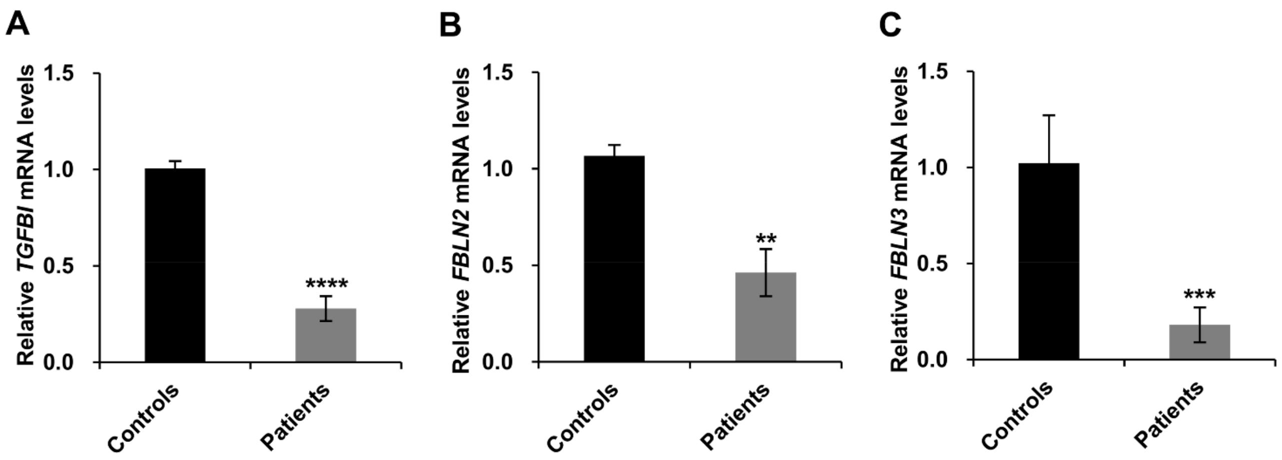

Supplementary Figure 5. The expression of microfibril and elastic fiber-associated molecules are downregulated in patients' skin fibroblasts. qRT-PCR analysis of *TGFBI*, *FBLN2*, and *FBLN3* expression in skin fibroblasts from controls (n=3) and patients (n=4). Data was expressed as means ± SD of three independent experiments. ** $p < 0.01$, *** $p < 0.001$, and **** $p < 0.0001$.

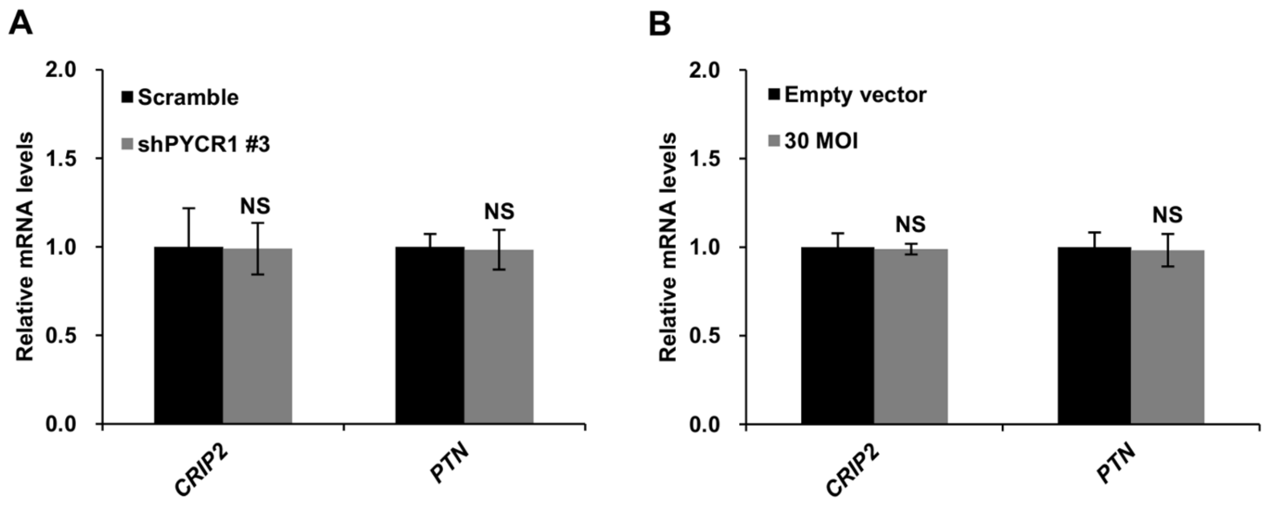

Supplementary Figure 6. The expression of *CRIP2* and *PTN* had no significant change in *PYCR1* knockdown or overexpressed skin fibroblasts. (**A**) qRT-PCR analysis of *CRIP2* and *PTN* expression in *PYCR1* knockdown (shPYCR1 #3, 2 MOI) skin fibroblasts. (B) qRT-PCR analysis of *CRIP2* and *PTN* expression in PYCR1 overexpressed skin fibroblasts. Data was expressed as mean±SD of three independent experiments. NS: not significant.

© 2018. Huang YW et al. Published online at http://www.aginganddisease.org/EN/10.14336/AD.2018.0222

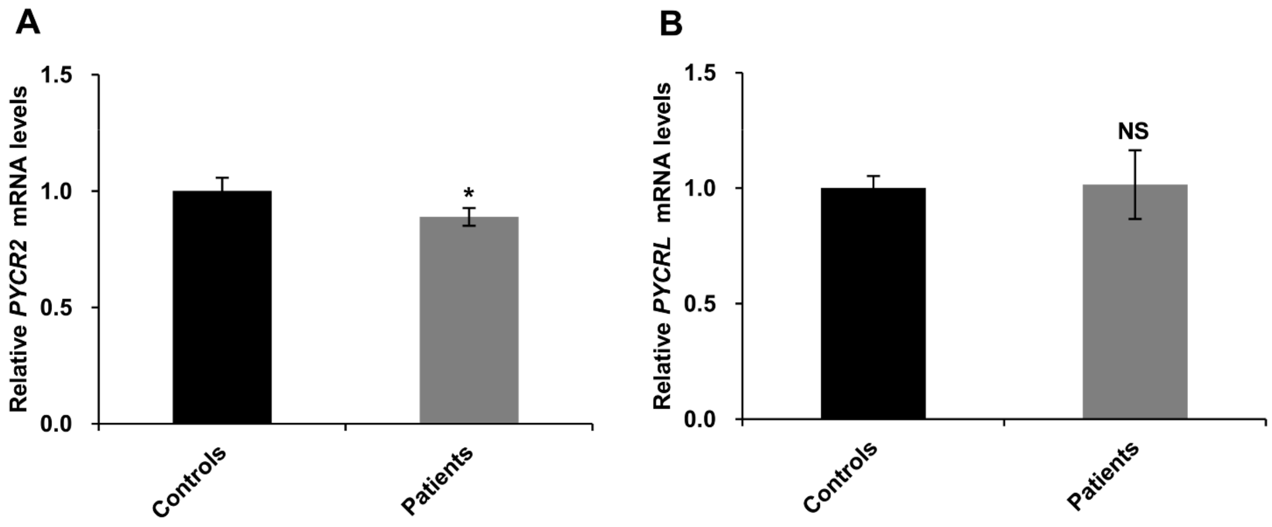

Supplementary Figure 7. The expression of *PYCR2* and *PYCRL* had no significant change in patients' skin fibroblasts. (**A**) **qRT-PCR analysis of** *PYCR2* gene expression in skin fibroblasts from controls (n=3) and patients (n=4). (**B**) qRT-PCR for *PYCRL* expression in skin fibroblasts from controls (n=3) and patients (n=4). Data was expressed as mean±SD of three independent experiments. NS: not significant; * $p < 0.05$.

© 2018. Huang YW et al. Published online at http://www.aginganddisease.org/EN/10.14336/AD.2018.0222
